# Depleted depletion drives polymer swelling in poor solvent mixtures

**DOI:** 10.1038/s41467-017-01520-5

**Published:** 2017-11-09

**Authors:** Debashish Mukherji, Carlos M. Marques, Torsten Stuehn, Kurt Kremer

**Affiliations:** 1 Max-Planck Institut für Polymerforschung, Ackermannweg 10, 55128 Mainz, Germany; 2 Institut Charles Sadron, Université de Strasbourg, CNRS, 23 rue du Loess, 67034 Strasbourg Cedex 2, France

## Abstract

Establishing a link between macromolecular conformation and microscopic interaction is a key to understand properties of polymer solutions and for designing technologically relevant “smart” polymers. Here, polymer solvation in solvent mixtures strike as paradoxical phenomena. For example, when adding polymers to a solvent, such that all particle interactions are repulsive, polymer chains can collapse due to increased monomer–solvent repulsion. This depletion induced monomer–monomer attraction is well known from colloidal stability. A typical example is poly(methyl methacrylate) (PMMA) in water or small alcohols. While polymer collapse in a single poor solvent is well understood, the observed polymer swelling in mixtures of two repulsive solvents is surprising. By combining simulations and theoretical concepts known from polymer physics and colloidal science, we unveil the microscopic, generic origin of this collapse–swelling–collapse behavior. We show that this phenomenon naturally emerges at constant pressure when an appropriate balance of entropically driven depletion interactions is achieved.

## Abstract (web summary)

Whilst polymer collapse in a single poor solvent is well understood, polymer swelling in mixtures of two repulsive solvents is surprising. Here, the authors unveil the microscopic origin of collapse-swelling-collapse behaviour, showing how it emerges at constant pressure when a balance of depletion interactions is achieved.

## Introduction

The understanding of coil-to-globule transition of a macromolecule in solvent mixtures is a fundamental process for functional soft matter with a huge variety of applications that goes beyond traditional polymer science^1, 2^. This reaches from the responsiveness of hydrogels to external stimuli^3, 4^ and biomedical applications^5–8^ to the processing of conjugated polymers for organic electronics^9^. In this context, it has been commonly observed that a polymer can collapse in a mixture of two competing, well miscible good solvents, while the same polymer remains expanded in these two individual components. This phenomenon is commonly known as co-non-solvency^2, 10–19^. However, it has also been observed that a polymer can be collapsed in two different poor solvents, whereas it is “better” soluble in their mixtures^20–23^. Thus far a multitude of specific, system-dependent explanations hindered the emergence of a clear physical picture of these two intriguing phenomena. While the phenomenon of co-non-solvency has been recently brought onto a firmer ground of a generic explanation^2, 18^, no equivalent understanding of the collapse–swelling–collapse behavior has yet been achieved.

In a standard poor solvent, starting from a good solvent condition, an increase of the effective attraction between the monomers first brings the polymer into Θ—conditions, where the radius of gyration scales as $R_{\mathrm{g}} \sim N_l^{1/2}$ with *N*_*l*_ being the chain length^24, 25^. Upon further increase of the attraction, a polymer then collapses into a globular state. The resultant collapsed globule can be understood by balancing negative second virial osmotic contributions and three-body repulsions. The effective attraction between the monomers of a polymer can be viewed as a depletion induced attraction, a phenomenon well described for colloidal suspensions^26–30^ of purely repulsive particles. In this context, monomer attraction will occur when monomer–solvent excluded volume interactions become large enough. The resulting isolated polymer conformation can be well described by the Porod scaling law of the static structure factor *S*(*q*) ∝ *q* ^−4^ following the envelope of the correlation peaks in *S*(*q*), presenting a compact spherical globule. Interestingly, even if a polymer exhibits poor solvent conditions in two different solvents, it can possibly be somewhat swollen by intermediate mixing ratios of the two poor solvents. A system that shows this collapse–swelling–collapse scenario is poly(methyl methacrylate) (PMMA) in aqueous alcohol mixtures. More specifically, water and alcohol are “almost” perfectly miscible and individually poor solvents for PMMA. However, PMMA shows improved solubility within intermediate mixing concentrations of aqueous alcohol and/or other solvent mixtures^20–23^.

In this work, we propose a microscopic, generic and thus quite generally applicable picture of this collapse–swelling–collapse behavior in poor solvent mixtures. Therefore, we aim to do the following: (1) devise a thermodynamically consistent generic (chemically independent) model such that solubility of many polymers in mixtures of poor solvents, including PMMA in aqueous alcohol, can be explained within a simplified (universal) physical concept, (2) develop a microscopic understanding of the collapse–swelling–collapse scenario and show that microscopically this is a second order effect, and (3) investigate if a polymer in mixed poor solvents can really reach a fully swollen state characteristic of good solvents. To achieve the above goals, we combine generic molecular dynamics, all-atom simulations and analytical theoretical arguments to study polymer behavior in poor solvent mixtures.

## Results

### Conformation of polymer

**Fig. 1:**
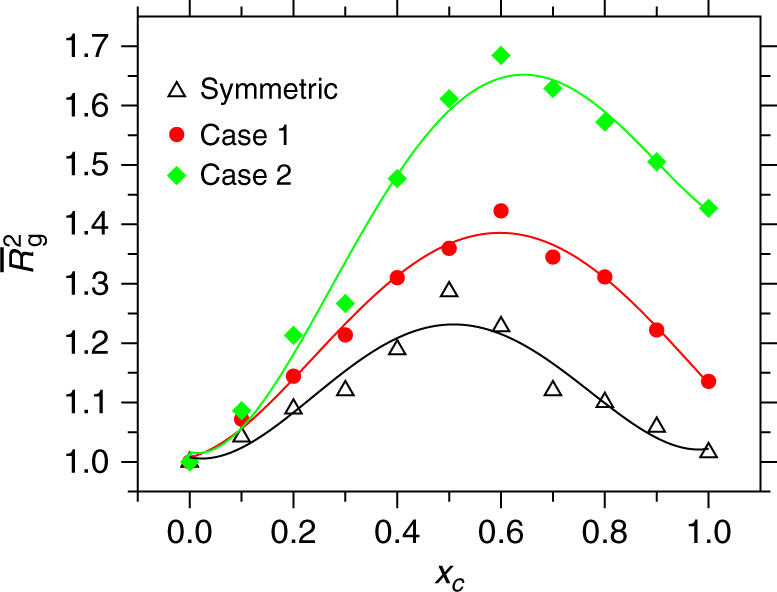
Polymer conformation and the effect of different mixtures of poor solvent conditions. Normalized squared radius of gyration $\overline R _{\mathrm{g}}^2 = \left\langle {R_{\mathrm{g}}^2} \right\rangle {\mathrm{/}}\left\langle {R_{\mathrm{g}}\left( {x_{\mathrm{c}} = 0} \right)^2} \right\rangle$ as a function of cosolvent molar concentration *x*_*c*_. Results are shown for the generic simulations and for three different cases. The parameter-specific details of the generic cases are listed in the Supplementary Table 1. The results are shown for a chain length of *N*_*l*_ = 30, which corresponds to $\sim 30\ell _p$ with $\ell _p$ being the persistence length. Here $\left\langle {R_{\mathrm{g}}\left( {x_{\mathrm{c}} = 0} \right)^2} \right\rangle$ = 2.6 ± 0.4*σ* ^2^ and $\overline R _\Theta ^2 = 2.13$ with $\overline R _\Theta = R_\Theta {\mathrm{/}}R_{\mathrm{g}}\left( {x_c = 0} \right)$ is the normalized Θ—point gyration radius. Here case 2 closely mimics the conformational behavior of PMMA in aqueous methanol mixture based on the parameterization presented in the Supplementary Table 1. Lines are polynomial fits to the data that are drawn to guide the eye

Figure 1 summarizes results for the normalized squared radius of gyration $\overline R _{\mathrm{g}}^2 = \left\langle {R_{\mathrm{g}}^2} \right\rangle {\mathrm{/}}\left\langle {R_{\mathrm{g}}\left( {x_{\mathrm{c}} = 0} \right)^2} \right\rangle$ as a function of cosolvent mole fraction *x*_*c*_ from the generic model and for three different cases described in the Supplementary Table 1. A closer look at the symmetric case of two almost perfectly miscible, but otherwise identical solvents (black Δ) shows that—while the pure solvent (*x*_*c*_ = 0) and the pure cosolvent (*x*_*c*_ = 1) are equally poor solvents for the polymer, the same polymer swells within the intermediate cosolvent compositions, reaching a maximum swelling of $\overline R _{\mathrm{g}}^2$ by ~20% at around *x*_*c*_ = 0.5. How could this be? Certainly, if both solvent and cosolvent were perfectly miscible, nothing should happen in this case, as this would be nothing but identical to a single component solution. Furthermore, given that this is a case of standard poor solvent collapse, polymer conformations are determined by depletion forces (or depletion induced attraction)^28^.

**Fig. 2:**
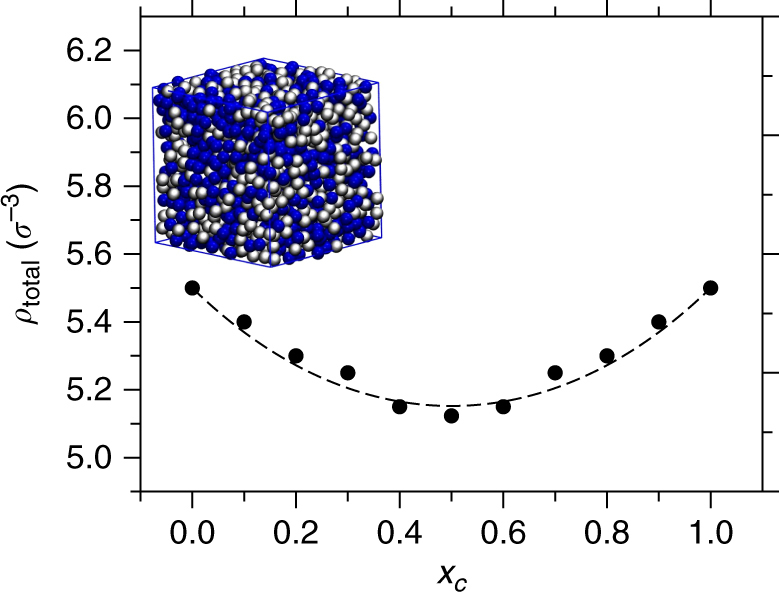
Dependence of bulk solution number density with different solvent–cosolvent mixing ratios. Main panel presents *ρ*_total_ for the generic model as a function of cosolvent mole fraction *x*_*c*_. The line is drawn based on Eq. (3), with *ρ*_total_ = 1/*v*. In the inset, we show a simulation snapshot of the generic system presenting bulk solution for a *x*_*c*_ = 0.5 mixture. The generic parameters for the bulk solution are chosen such that the density dip observed in generic model closely resembles aqueous methanol mixture, see Supplementary Fig. 1

When cosolvents are added into the polymer–solvent system (such as the addition of alcohol in a PMMA–water system), the addition of cosolvents not only repels (or depletes) monomers, but also repels solvents and vice versa. In this context, if we analyze the all-atom system of aqueous alcohol mixtures, we find that the total number density of the system *ρ*_total_ shows a minimum at 50/50 mixing ratios at constant pressure of 1 atm and temperature of 300 K, see Supplementary Fig. 1. This suggests that, when alcohol is added in water, the repulsive forces between the solution components result in a dip in *ρ*_total_, which is also known from experiments^31^. In our generic simulations, we tune solvent–solvent, solvent–cosolvent, cosolvent–cosolvent interactions, temperature *T* and pressure *P* such that we reproduce the density dip observed in the all-atom simulations. Furthermore, the system parameters are chosen such that the bulk solvent–cosolvent solution mixture remains deep in the miscible state far from phase separation. The representative simulation snapshot is shown in Fig. 2 for a 50/50 solvent–cosolvent mixture. In the main panel of Fig. 2, we show *ρ*_total_ used in our generic simulations. It can be seen that, in the generic model, we also find a density dip of about 10% at *x*_*c*_ = 0.5, which is consistent with the all-atom simulations. This leads to an effectively reduced repulsive interaction around *x*_*c*_ = 0.5 because of the reduced number of solvent particles near the monomer as expected from the variation of *ρ*_total_ with *x*_*c*_. The net result is a swelling of the polymer chain around *x*_*c*_ = 0.5. We coin here the term depleted depletion for explaining the reduction of depletion forces responsible for polymer collapse due to mutual solvent–cosolvent exclusion. Notice, however, that this is a common concept in colloidal science, where the modifications of the depletion attraction profile due to depletant-depletant interactions have been extensively studied^28–30^.

When the interaction asymmetry between polymer-cosolvent *ε*_*pc*_ and polymer–solvent *ε*_*ps*_ is increased (Supplementary Table 1), where *ε*_*pc*_ for case 2 < case 1 < symmetric case, not only the degree of swelling increases, but the swelling region also shifts between 0.5 < *x*_*c*_ < 0.9. This range is found to be in excellent agreement with the experimental observation of PMMA conformations in aqueous alcohol mixtures^21, 22^. Specifically, our case 2 closely resembles PMMA in an aqueous methanol mixture. Indeed, we tune our monomer–solvent and monomer–cosolvent interactions in the generic model such that we can reproduce the correct solvation free energy, as measured by the shift in excess chemical potential per monomer $\overline {\mu _{\mathrm{p}}}$, known from all-atom simulations of a PMMA system in aqueous methanol mixtures (Supplementary Fig. 4 and Supplementary Note 2). Furthermore, because we reproduce $\overline \mu _{\mathrm{p}}$ and *ρ*_total_ variation with changing *x*_*c*_ in our generic model as known from all-atom simulations under ambient condition, *T* = 0.5*ε*/*k*_B_ in the generic model corresponds to 300 K and *P* = 16.0*ε*/*σ* ^3^ corresponds to 1 atm in all-atom system. While the swelling around $x_{\mathrm{c}} \sim 50\%$, especially for the symmetric case, is bulk solution number density dependent (at constant pressure), the shift in the region of maximal swelling is cosolvent–monomer interaction dependent. For example, cosolvent–monomer repulsion for symmetric case > case 1 > case 2. This is similar to the PMMA solvation in different aqueous alcohol mixtures, where the repulsion of methanol–MMA > ethanol–MMA > propanol–MMA^20–23^.

### Single-chain structure factor

**Fig. 3:**
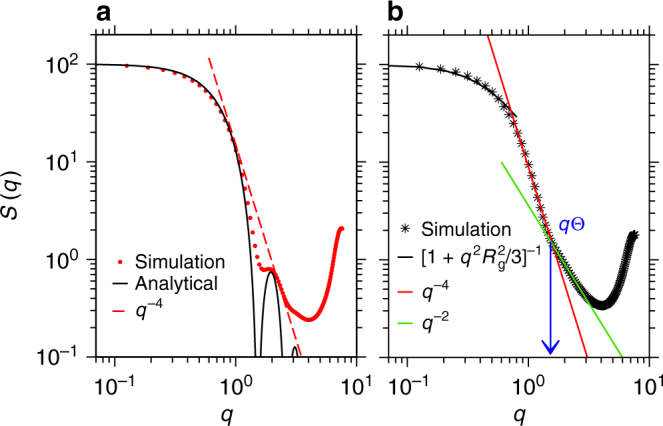
Single-chain structure factor. Static structure factor *S*(*q*) for a chain of length *N*_*l*_ = 100. **a** *S*(*q*) at *x*_*c*_ = 0.0 and (**b**) *x*_*c*_ = 0.7. In **a**, we also include the analytical expression for sphere scattering. In **b**, red and green lines are power law fits to the data at different length scales. The black line represents the Guiner region for *q*→0 (for large length scales). The vertical arrow indicates the effective Θ—blob size at *q* = *q*_Θ_, estimated using $\ell _{\Theta - {\mathrm{blob}}} = 2\pi {\mathrm{/}}q_\Theta$. Note: to get a better estimate of the cross-over scaling regime, *S*(*q*) is calculated from a simulation of a chain length *N*_*l*_ = 100

A closer look at Fig. 1 shows that the degree of swelling, within the range 0.5 < *x*_*c*_ < 0.9, varies between 20 and 65% (or 10 and 30% in $\overline R _{\mathrm{g}}$), depending on the interaction assymetry. Considering that we are dealing with combinations of poor solvents, this is a very significant swelling, making PMMA-based materials permeable to water–alcohol mixtures. Moreover, analyzing the simulations, it becomes apparent that the polymer does not necessarily reach a fully swollen configuration. A quantity that perhaps best characterizes a polymer conformation is the polymer form factor *S*(*q*). In Fig. 3, we present *S*(*q*) for two different values of *x*_*c*_ for the system described by case 1. Part (a) shows *S*(*q*) of a fully collapsed chain in pure solvent (*x*_*c*_ = 0) and part (b) presents maximum polymer swelling (*x*_*c*_ = 0.7). For *x*_*c*_ = 0.0, the polymer can be well described by a scaling law known for sphere scattering (Porod scattering), namely $S(q) \sim q^{ - 4}$ following the envelope of *S*(*q*) curve, suggesting a fully collapsed poor solvent conformation. Furthermore, the data point corresponding to *x*_*c*_ = 0.7 shows more interesting polymer conformations. Within the range 1.5*σ* ^−1^ < *q* < 3.0*σ* ^−1^, an aparent scaling $S(q) \sim q^{ - 2}$ is observed, which crosses over to $S(q) \sim q^{ - 4}$ for 0.7*σ* ^−1^ < *q* < 1.5*σ* ^−1^, suggesting that the polymer remains globally collapsed, consisting of Θ—blobs. The cross-over point *q*_Θ_ gives the direct measure of the effective blob size $\ell _{\Theta - {\mathrm{blob}}} = 2\pi {\mathrm{/}}q_\Theta \sim 4.5\sigma$. The largest blobs are observed when the polymer is maximally swollen.

## Discussion

**Fig. 4:**
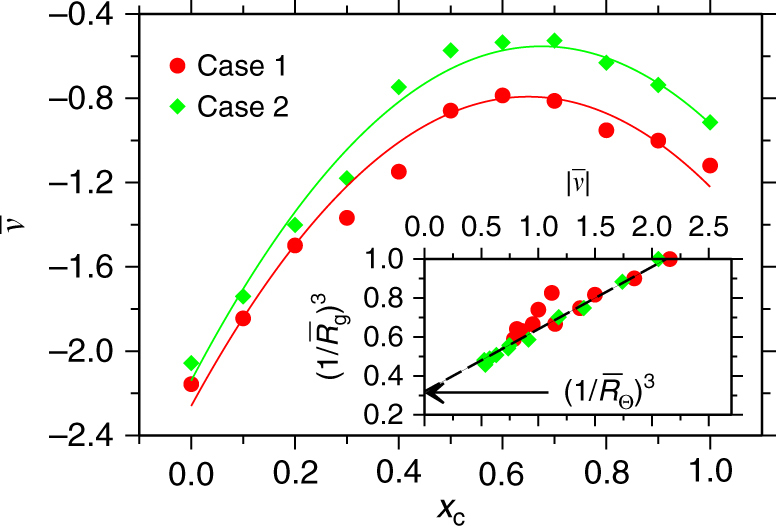
Variation in monomer excluded volume with changing solvent–cosolvent mixing ratios. Normalized excluded volume $\overline {\cal V} = {\cal V}{\mathrm{/}}{\cal V}_{\mathrm{m}}$ as a function of cosolvent mole fraction *x*_*c*_. The results are shown for two cases. Lines are fits to the data corresponding to Eq. (1). In the inset, we present $\left( {1{\mathrm{/}}\overline R _{\mathrm{g}}} \right)^3$ as a function of normalized ${\cal V}$. Here, $\overline R _{\mathrm{g}} = R_{\mathrm{g}}{\mathrm{/}}R_{\mathrm{g}}\left( {x_{\mathrm{c}} = 0} \right)$ is the normalized gyration radius *R*_g_. The line is a fit based on Eq. (5)

So far we have discussed that the collapse–swelling–collapse scenario naturally emerges because of the constant pressure. However, when the number densities for different mixing ratios are kept fixed (such that the pressure rises within the intermediate solvent–cosolvent mixing), the collapse–swelling–collapse scenario is not observed. Only when *ρ*_total_ is allowed to vary with changing solvent–cosolvent molar composition (as shown in Fig. 2), we can observe swelling of a polymer. Therefore, we now describe the observed collapse–swelling–collapse phenomenon within the mean-field level by the Flory–Huggins (FH) theory and its variants. For the case where a polymer with chain length *N*_*l*_, at volume fraction *ϕ*_p_, is dissolved in a mixture of two components *s* and *c*, respectively, FH theory predicts a monomer–monomer excluded volume of the form^24, 25^,

1 $$\overline {\cal V} = 1 - 2\left( {1 - x_{\mathrm{c}}} \right)\chi _{{\mathrm{ps}}} - 2x_{\mathrm{c}}\chi _{{\mathrm{pc}}} + 2x_{\mathrm{c}}\left( {1 - x_{\mathrm{c}}} \right)\chi _{{\mathrm{sc}}},$$

where *χ*_*ps*_ and *χ*_*pc*_ are the Flory–Huggins interaction parameters between *p* − *s* and *p* − *c*, respectively. The factor *χ*_*sc*_ is the parameter of *s* − *c* interaction. When both solvent and cosolvent are poor solvents, *χ*_*ps*_ > 1/2 and *χ*_*pc*_ > 1/2. In our simulations, $\overline {\cal V} = {\cal V}{\mathrm{/}}{\cal V}_{\mathrm{m}}$ is calculated using the expression ${\cal V} = 2\pi {\int} \left[ {1 - e^{ - v(r)/k_{\mathrm{B}}T}} \right]r^2{\mathrm{d}}r$. We use *v*(*r*) = −*k*_B_ *T* ln[g(*r*)] as a guess of the potential of mean force (PMF), which is calculated from the radial distribution function between non-bonded monomers g(*r*). ${\cal V}_{\mathrm{m}} = 2.73 \sigma ^3$ is the bare monomer excluded volume in the absence of any (co)solvent and corresponds to a monomer–monomer distance of 0.87*σ*. Fitting Eq. (1) to the $\overline {\cal V}$ data in Fig. 4, we find *χ*_*ps*_ = 1.57, *χ*_*pc*_ = 1.11, and *χ*_*sc*_ = 1.74 for case 1 and *χ*_*ps*_ = 1.62, *χ*_*pc*_ = 0.95, and *χ*_*sc*_ = 1.74 for case 2. Consistently, *χ*_*sc*_ values for both cases are similar and independent of polymer–solvent interactions. Here, it is important to note that the $\overline {\cal V}$ values in Fig. 4 were obtained in simulations that were performed when the bulk solution density varies over full *x*_*c*_, keeping pressure invariant. Therefore, the *χ* values obtained are not related to constant density case. Furthermore, the value of *χ*_*sc*_ obtained here is consistent with the experimental value obtained in ref.^11^, but might appear as too strong considering that one is dealing with well miscible solvents. However, this is simply a consequence of performing the analysis with free-energy densities normalized by the monomer volume, a natural choice to inspect polymer collapsing behavior. Within this normalization, however, *χ*_*sc*_ parameter would corresponds to the effective interaction between two clusters consisting of solvent and cosolvent particles. Therefore, if one would consider exclusively the solvent–cosolvent system, the natural choice for normalizing free-energy densities would be the excluded volume of the solvents that are about eight times smaller than that of ${\cal V}_{\mathrm{m}}$, and that would lead, in those units, to a value of $\chi _{{\mathrm{sc}}}^ \star \sim \chi _{{\mathrm{sc}}}{\mathrm{/}}8 \sim 0.22$. Note also that standard FH predictions assume that solvent and cosolvent because of the fixed grid size in the lattice—and also polymer—are mixed at constant volume, whereas our simulations just as the experiments are performed at a constant pressure. It should also be noted that the constant density FH theory put forward in ref.^19^ requires a strong repulsive interaction parameter between solvent and cosolvent making $\chi \gg k_{\mathrm{B}}T$, but in common mixtures of water and alcohol $\chi \sim k_{\mathrm{B}}T$. Therefore, in the following, we derive a FH expression for the ${\cal V}$ values at constant *p*, which predicts effective values for *χ*_*sc*_ dependent on *p*. The subtle interaction details seen here are not only restricted to polymer solutions, but are also important for sequence dependent miscibility of copolymers^32, 33^.

**Fig. 5:**
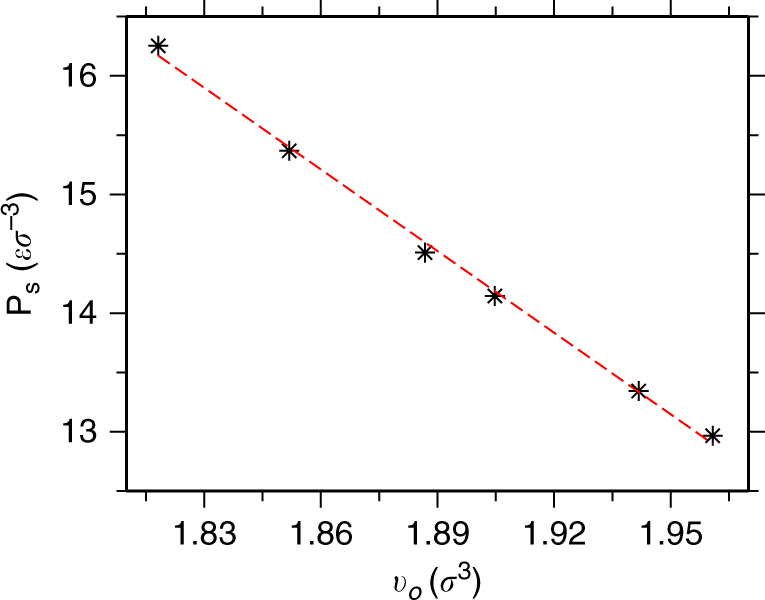
Pressure change in bare solvent with the change in molar volume. Variation of pressure *P*_*s*_ with change in molar volume *v* for a bare solvent system

In our simulations, we only consider polymer under infinite dilution *ϕ*_*p*_ → 0 and the large majority of the volume is occupied by solvent–cosolvent mixture. Therefore, we concentrate our analysis on the binary mixture. Additionally, we also consider that the pure reference solvent and cosolvent systems are identical, but that *s*−*c* interactions are distinct from those for *s*−*s*, and *c*−*c*. For this case, the total free energy is written as

2 $$\begin{array}{ccccc}\\ \frac{{{\cal F}v}}{{\kappa _{\mathrm{B}}T}} = & \frac{{v{\cal F}_s(v)}}{{\kappa _{\mathrm{B}}T}} + x_{\mathrm{c}}\mathrm{ln}\left( {{\it x}_{\mathrm{c}}} \right) + \left( {1 - {\it x}_{\mathrm{c}}} \right)\mathrm{ln}\left( {1 - {\it x}_{\mathrm{c}}} \right),\\ \\ & + \chi _{{\mathrm{sc}}}(v)x_{\mathrm{c}}\left( {1 - x_{\mathrm{c}}} \right),\\ \end{array}$$

where ${\cal F}_{\mathrm{s}}(v)$ is the volume-dependent free-energy of the pure (co)solvent systems and where we consider the explicit dependence of *χ*_*sc*_ on system volume. Note that, since experiments and simulations are performed at constant number of molecules ${\cal N}$, the total volume of the system *V* is simply given by $V = {\cal N}v$. For a given external pressure *P*, the molar volume *v* is thus controlled by, *P* = *P*_*s*_(*v*) − *κ*_B_ *Tx*_*c*_(1 − *x*_*c*_)∂*χ*_*sc*_(*v*)/∂*v* with $P_{\mathrm{s}}(v) = - \partial v{\cal F}_{\mathrm{s}}{\mathrm{/}}\partial v$, being the pressure of the reference system. If one assumes a small variation of the molar volume of the solvent–cosolvent mixture with respect to that of the reference system, one gets

3 $$v = v_{\mathrm{o}}\left[ {1 + \zeta \;x_{\mathrm{c}}(1 - x_{\mathrm{c}})} \right],$$

where *ζ* = *κ*_B_ *T*/*v* ∂*χ*_*sc*_(*v*)/∂*v*[∂*P*_*s*_(*v*)/∂*v*]^−1^ measures the relative sensitivity of the interaction parameter and reference pressure to *v*. In Fig. 5, we show *P*_*s*_ as a function of *v* that gives an estimate of ∂*P*_*s*_(*v*)/∂*v* = 20*ε*/*σ* ^6^. Equation (3) describes well the observed density variation of the generic model in Fig. 2 with *ζ* = 0.26. Note that *ρ*_total_ and molar volume *v* are simply related by *ρ*_total_ = 1/*v*. Also, to first order in (*v*−*v*_o_)/*v*_o_, which for our generic model is of the order of 10%, one gets

4 $$\chi _{{\mathrm{sc}}}(v) = \chi _{{\mathrm{sc}}}\left( {v_{\mathrm{o}}} \right) + v\left. {\frac{{\partial \chi _{sc}(v)}}{{\partial v}}} \right|_{x_{\mathrm{c}} \to 0}\zeta x_{\mathrm{c}}\left( {1 - x_{\mathrm{c}}} \right).$$

Since $v\partial \chi _{{\mathrm{sc}}}(v){\mathrm{/}}\partial v \sim \zeta$, this shows that *χ*_*sc*_ obtained between different ensembles is only perturbed to the second order in *ζ*. This will lead to an effective expression *χ*_*sc*_(*v*) = *χ*_*sc*_(*v*_o_) − 0.096*x*_*c*_(1 − *x*_*c*_). Furthermore, if we choose *x*_*c*_ = 0.5, the above equation will lead to a ~11% variation in *χ*_*sc*_ values with respect to the standard values calculated when *ρ*_total_ is kept constant. This suggests that effective *χ*_*sc*_ obtained in different ensembles is rather close. Therefore, showing that, in this system, *χ*_*sc*_ parameter relevant to the FH analysis keeps consistent values throughout the range of compositions. It should also be mentioned that the variation in *χ*_*sc*_ is a result of bulk density variation at *x*_*c*_, which is about 10% (see Fig. 2). Moreover, we find that when the density is kept constant, there is no variation of polymer conformation over full *x*_*c*_ range, while pressure of the system goes up with a maximum at *x*_*c*_ = 0.5.

Our numerical predictions successfully account for polymer swelling in solutions of poor solvent mixtures, as the simulations quantitatively demonstrate. While this fascinating polymer behavior is driven by purely repulsive interactions, it also reveals the subtle balance of depletion forces and bulk solution properties that enable such a paradoxical phenomenon. Indeed, polymer collapse in repulsive solvents can be understood by depletion induced attractions^28^. The dominant contribution to depletion induced attraction originates from direct monomer–solvent repulsion, and is thus proportional to solvent number density *ρ*_totat_ dictating the number of depletants. When a few solvent molecules are replaced by cosolvents, for example, a water by an alcohol, this preserves the solvent density to the first order. Under these conditions, one smoothly interpolates between two polymer collapsed states, without any swelling at intermediate compositions. Here, however, interactions between solvent components play a delicate role in dictating the depletion forces by bringing in contributions proportional to second (or to even higher)-order contributions of *ρ*_totat_, see Fig. 2. Interestingly, it is well known from colloidal sciences that such second order effects may reduce colloid–colloid attractive forces^29, 30^. Moreover, these studies in colloidal systems typically deal with two component systems where size asymmetry of 10 is needed to observe higher order effects. In our study, size asymmetry between monomer and solvent is significantly smaller and second order effects originate because of the peculiar properties of the solvent–cosolvent mixtures. Thus, the polymer case occurs in a different interaction regime compared to colloidal effect. The solvent–cosolvent excluded volume is slightly stronger than the corresponding values for solvent–solvent and cosolvent–cosolvent molecules, leading to a slightly smaller solution density and a corresponding diminution of the effective depletion interaction. At intermediate compositions, where solvent–cosolvent interactions are dominant in the solution, the effect is the strongest. Therefore, a broad variety of polymer/solvent systems are expected to display such a behavior.

A standard measure of the attractive forces leading to polymer collapse is provided by the monomer excluded volume ${\cal V}$. For poor solvents, ${\cal V}$ is negative and the dimensions of the chain can be understood by balancing the second (negative) virial osmotic contributions and the three body repulsion^24, 25^, leading to

5 $$\frac{{\overline R _\Theta ^3}}{{\overline R _{\mathrm{g}}^3}} - 1 = \left| {\overline {\cal V} } \right|.$$

In the inset of Fig. 4, we show $\left( {1{\mathrm{/}}\overline R _{\mathrm{g}}} \right)^3$ as a function of $\overline {\cal V}$, where the *R*_g_ is taken from Fig. 1a and $\overline {\cal V}$ is given by the values in the main panel of Fig. 4. The data are well described by the theoretical prediction in Eq. (5). Extrapolating the data to ${\cal V} = 0$, we estimate $\overline R _\Theta = 1.46$ (or *R*_Θ_ = 2.34*σ*), further suggesting that the polymer remains below Θ—conformation, even when it swells within intermediate mixing ratios.

This collapse–swelling–collapse scenario of PMMA in aqueous alcohol appears as the opposite effect to that of coil-globule-coil scenario, e.g., PNIPAm in aqueous alcohol, often referred to as co-non-solvency^2, 11, 12^. However, the coil-globule-coil transition occurs when individually good, but competing, solvents for a polymer are mixed together and as a result polymer collapses within the intermediate mixing ratios. Because this is microscopically a good solvent system, it is dictated by the competition between solvent and cosolvent preferential adsorption with the polymer^2, 34^. Typical systems where co-nonsolvency is observed, require an interaction contrast of about 4*k*_B_ *T* ^17^. Therefore, a small change (i.e., ~10%) in bulk solution density does not significantly influence the polymer conformation. On the contrary, the collapse–swelling–collapse behavior, studied here, is due to a subtle balance of repulsive microscopic interactions and the bulk solution density. Furthermore, our analysis also suggests that the collapse–swelling–collapse sequence in poor solvent mixtures is driven by the mean-field behavior, i.e., contrary to the co-nonsolvency effect that can not be described by a Flory–Huggins mean-field picture because of the strong enhancement of the cosolvent concentration in the vicinity of the polymer chain^18^. Here, the solvent–cosolvent interaction parameter *χ*_*sc*_, though quite small, plays a key role. Our results clarify that although collapse–swelling–collapse and co-nonsolvency appear as two symmetric manifestations of polymer solubility, they are in fact driven by markedly different physical mechanisms.

In conclusion, we have performed molecular dynamics simulations to unveil the microscopic origin of polymer swelling in poor solvent mixtures. We propose a unified generic picture of the polymer collapse–swelling–collapse phenomenon. This conformational change is due to a delicate balance between the depletion forces and the bulk solution density at constant pressure. Combining the Flory–Huggins type mean-field picture with molecular dynamics simulations, we show that the polymer swelling in poor solvents is dictated by reduced depletion forces that originate from the bulk solution properties. These results show semi-quantitative agreement of the polymer swelling behavior in mixtures of two miscible poor solvents such as the solvation of PMMA in aqueous alcohol mixtures. While the polymers swell significantly, the mostly swollen polymer structure still remains below Θ—conformation. Even when we take PMMA as a test case, there are systems, such as corn starch^35^ and poly(*N*-(6-acetamidopyridin-2-yl)acrylamide)^36^, which also show collapse–swelling–collapse behavior. Interestingly, the solvent–cosolvent mixtures in these cases are also aqueous alcohol mixtures. Further supporting that the delicate balance between microscopic repulsion together with density dip of the bulk solution plays a key role in describing this phenomenon. Being potentially applicable to a large variety of polymers, the concepts presented here may pave ways towards the functional design of “smart” polymeric systems for advanced biomedical purposes.

## Methods

All generic simulations are based on the “well-known” bead-spring model of polymers^37^. In this model, individual monomers of a polymer interact with each other via a repulsive 6–12 Lennard–Jones (LJ) potential with a cutoff *r*_*c*_ = 2^1/6^ *σ*. Additionally, adjacent monomers in a polymer are connected via a finitely extensible nonlinear elastic potential (FENE). The parameters are chosen such that a reasonably large time step can be chosen. The results are presented in units of the LJ interaction energy *ε*, LJ length unit *σ* and unit of mass *m*. This leads to a time unit of *τ* = *σ*(*m*/*ε*)^1/2^.

A bead-spring polymer *p* is solvated in mixed solutions composed of two components, solvent *s* and cosolvent *c*, respectively. The mole fraction of the cosolvent component *x*_*c*_ is varied from 0 (pure *s* component) to 1 (pure *c* component). The size of monomers is *σ*_*p*_ = 1.0*σ* and sizes of the (co)solvent molecules are chosen as *σ*_*s/c*_ = 0.5*σ*. This specific choice of *σ*_*s/c*_ is due to the fact that the monomers are typically twice the size of solvent molecules such as water and smaller alcohol. Because *s* and *c* are both individually poor solvents for the polymer, *p*–*s* and *p*–*c* interactions are always repulsive LJ with a cutoff *r*_*c*_ = 2^1/6^ *σ*_ij_, where *σ*_*ij*_ is the diameter of *p*–*s* and *p*–*c* interactions given by the combination rule *σ*_*ij*_ = (*σ*_*i*_ + *σ*_*j*_)/2. Here we choose, *σ*_*pp*_ = 1.0*σ*, *σ*_*ps*_ = 0.75*σ*, *σ*_*pc*_ = 0.75*σ*, *σ*_*ss*_ = 0.50*σ*, *σ*_*cc*_ = 0.50*σ*, and *σ*_*sc*_ = 0.50*σ*. We consider two different cases of solvent qualities that are dictated by the pairwise *ε*. A detailed description of *ε* between the LJ interaction energies of the individual “pure” poor (co)solvents are presented in Supplementary Table 1.

We consider a chain of length *N*_*l*_ = 30 solvated in a mixture of 2.0 × 10^4^ solution particles, in some cases, we also use *N*_*l*_ = 100 solvated in 5.0×10^4^ solution particles. The interactions between different solvent particles are all chosen as repulsive LJ to mimic depletion effects, as in the case of standard poor solvent collapse. LJ interaction energies *ε* are chosen to match the typical thermodynamic condition known from all-atom simulations. The equations of motion are integrated using a velocity Verlet algorithm with a time step *δt* = 0.01*τ*. The simulations were usually equilibrated for 10^7^ MD time steps. The measurements are typically observed over another 10^6^ MD steps. During this time, observables such as the gyration radius *R*_g_, static structure factor *S*(*q*), chemical potential of polymer *μ*_*p*_, and the polymer excluded volume ${\cal V}$ is calculated. The temperature is set to 0.5*ε*/*k*_B_ *T*, which is employed using a Langevin thermostat with damping constant *γ* = 1.0*τ* ^−1^.

One of the most important aspects of modeling PMMA in aqueous alcohol is to incorporate bulk solution properties. As mentioned earlier in the main manuscript text, alcohol and water are poor solvents for PMMA, while it swells in water–alcohol mixtures. Analyzing the experimental data^31^ and all-atom simulations of aqueous alcohol mixtures, it has become apparent that the excess volume of the mixtures increases (or decrease in the total solution number density *ρ*_total_) from the mean-field values, which follows in a nonlinear dependence with *x*_*c*_ (Supplementary Fig. 1). This deviation is most dominant at intermediate mixing ratios. In our generic simulation protocol, we choose interaction parameters of the solution components such that *ρ*_total_ of the solution decreases at around 50–50 mixture, while keeping the solution at constant pressure. For this purpose, we choose *ε*_*ss*_ = *ε*_*cc*_ = 0.5 and *ε*_*sc*_ = 2.5, keeping all the interactions repulsive (Supplementary Table 1). It is important to mention that *ρ*_total_ = 5.5*σ* ^−3^ for pure *x*_*c*_ = 0 and *x*_c_ = 1 solutions. This corresponds to a pressure of *p* ≈ 16.0 ± 0.5*ε*/*σ* ^3^. The advantage of this choice of *ρ*_total_ is that the solution remains stable over full *x*_*c*_ range, when the *ρ*_total_ decreases by ≈10% at *x*_*c*_ = 0.5.

We also want to mention that even when the parameters are chosen as repulsive with *c*−*s* being more repulsive than *c*−*c* and *s*−*s* interaction, our bulk solution remains homogeneous over the full range of mixing ratios. In this context, it is important to note that the solution phase separation is intimately linked to the solution density. Within our choice of *ρ*_total_, we do not see any phase separation. In Supplementary Fig. 2, we show three simulation snapshots for different *ρ*_total_ and for *x*_*c*_ = 0.5. It can be appreciated that there is no signature of phase separation when *ρ*_total_ = 5.2*σ* ^−3^, phase separation can only be seen for $\rho _{{\mathrm{total}}}\, \gtrsim \, 6.4\sigma ^{ - 3}$. Suggesting that the bulk solution remains stable. Furthermore, to quantify the possibility of any phase separation we calculate the quantity *η* defined as,

6 $$\eta = {\cal V}_{{\mathrm{ss}}} + {\cal V}_{{\mathrm{cc}}} - 2{\cal V}_{{\mathrm{sc}}}.$$

Here ${\cal V}_{ij}$ is the excluded volume of the *i*−*j* interaction defined as,

7 $${\cal V}_{ij} = 2\pi {\int}_0^\infty \left[ {1 - e^{ - v_{ij}(r)/\kappa _{\mathrm{B}}T}} \right]r^2{\mathrm{d}}r,$$

where *v*_*ij*_ is the potential of mean force between *i* and *j* components. We find $\eta = - 0.4\sigma ^3$ for *ρ*_total_ = 5.2*σ* ^−3^, *η* = −5.5*σ* ^3^ for *ρ*_total_ = 6.4*σ* ^−3^, and *η* < −30.0*σ* ^3^ for *ρ*_total_ = 8.0*σ* ^−3^. It can be appreciated that *η*→0 for *ρ*_total_ → 5.2*σ* ^−3^, further suggesting that the bulk solution is stable.

The details about generic simulations and all-atom force field parameters are given in the electronic Supplementary Material. Generic simulations are performed using the ESPResSo +  + molecular dynamics package^38^, all-atom simulations are performed using the GROMACS package^39^, and simulation snapshots are rendered using VMD^40^.

### Data availability

All presented and analyzed the data is included in both main text and in the Supplementary Material, including the methods, force fields, and theory developed. Generic simulation scripts will be made available through the ESPResSo++ webpage http://www.espresso-pp.de/.

## Electronic supplementary material

Supplementary Information
