# Supplementary material for: Depleted depletion drives polymer swelling in poor solvent mixtures
Source: Nat Commun. 2017 Nov 9;8:1374. doi: 10.1038/s41467-017-01520-5 (PMC5680348; doi:10.1038/s41467-017-01520-5)
Supplement: Supplementary file 1 — Supplementary Information [file 41467_2017_1520_MOESM1_ESM.pdf]

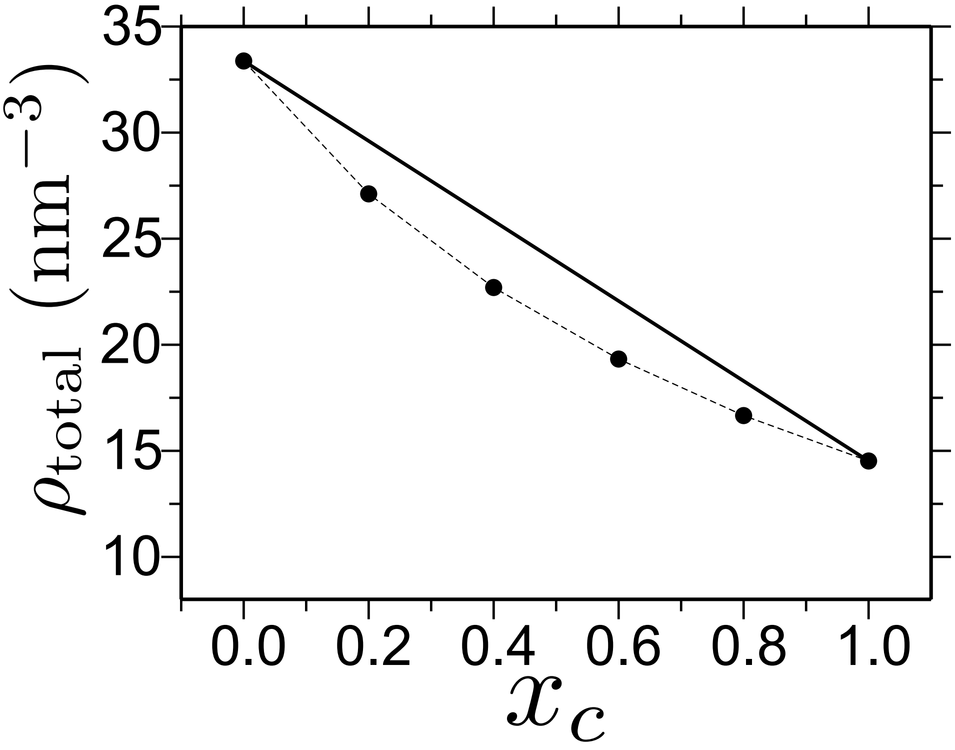

Supplementary Figure 1: Total number density ρ_{total} of the bulk solution as a function of cosolvent mole fraction x_c for aqueous methanol solution for ambient pressure and 298 K temperature. The data is extracted from the all-atom simulations. Solid lines are linear interpolation between the data points of $x_c = 0.0$ and $x_c = 1.0$.

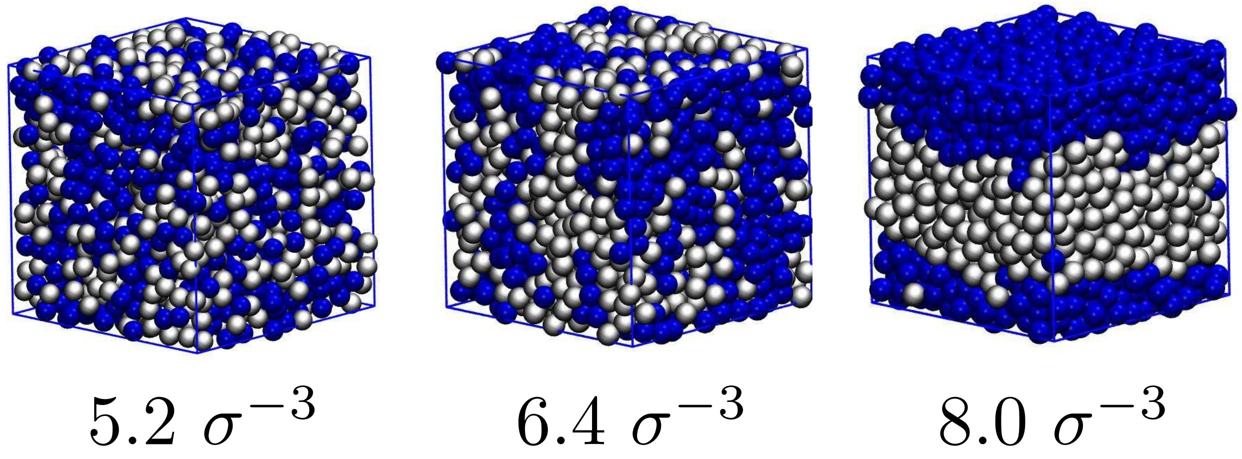

Supplementary Figure 2: Simulation snapshots of the generic system presenting bulk solution arrangements for three different densities. The results are shown for $x_c = 0.5$.

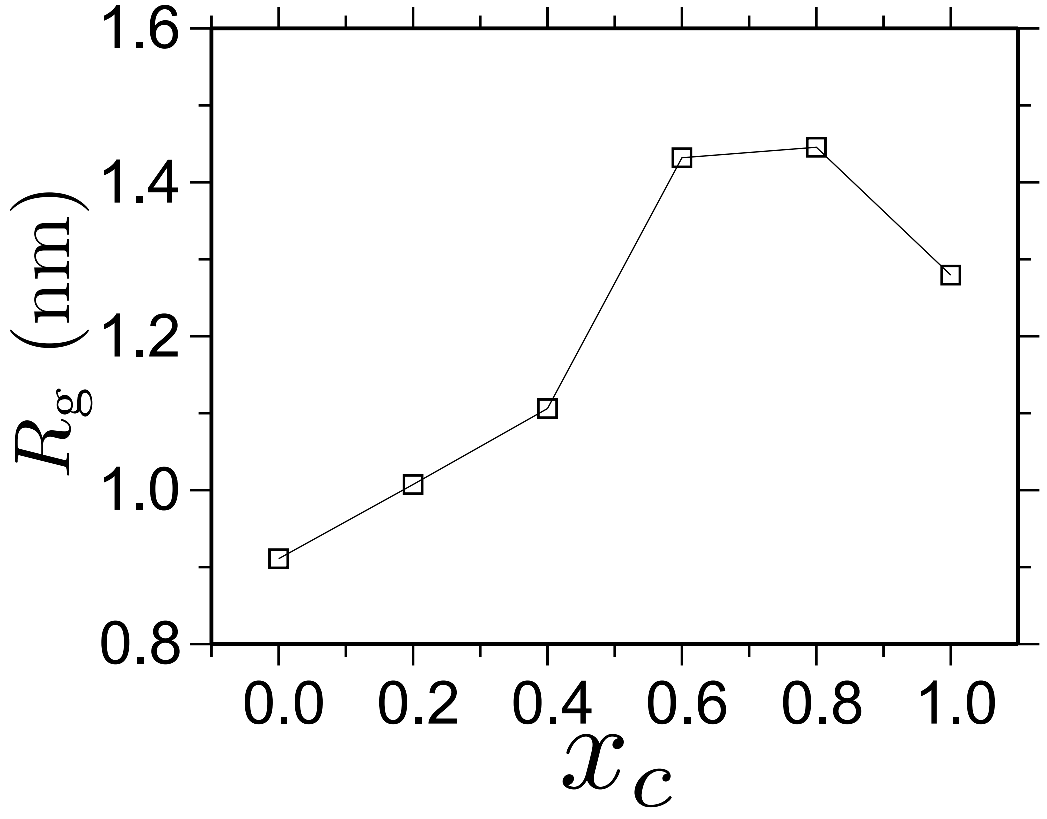

Supplementary Figure 3: Gyration radius R_{g} of a PMMA chain as a function of methanol mole fraction x_c.

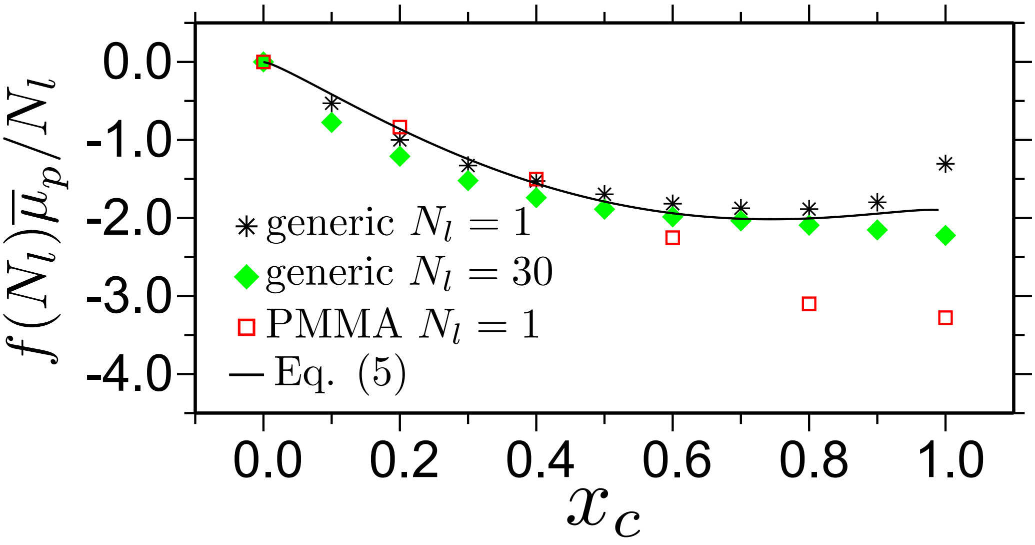

Supplementary Figure 4: Chemical potential shift $\overline{\mu}_p = \mu_p/k_{\mathrm{B}}T$ per monomer as a function of cosolvent mole fraction x_c. The master curve is obtained by normalizing $\overline{\mu}_p$ with a chain length N_l dependent function $f(N_l) = 2N_l/(N_l+1)$. The line is a fit to the data by Supplementary Eq. 2.

Supplementary Table 1: Lennard-Jones (LJ) interactions for the generic model. p, s and c represents polymer, solvent and cosolvent, respectively.

LJ energy	Symmetric	Case 1	Case 2	Cut-off
ϵ_{pp}	1.0ϵ	1.0ϵ	1.0ϵ	$2^{1/6}\sigma$
ϵ_{ps}	3.5ϵ	3.5ϵ	3.5ϵ	$0.75 \times 2^{1/6}\sigma$
ϵ_{pc}	3.5ϵ	2.5ϵ	2.0ϵ	$0.75 \times 2^{1/6}\sigma$

Supplementary Note 1: Computational details

In this work a modified OPLS force field of methyl acetate [1] was used to simulate PMMA. We use the SPC/E water model [2] and OPLS force field for methanol [3]. A more detailed analysis of the all-atom force field will be presented elsewhere [4].

The temperature is set to 300 K using a Berendsen thermostat with a coupling constant 0.1 ps. The time step for the simulations is chosen as 1 fs. To obtain equilibrium solvent density, initial configurations are equilibrated for 5 ns using a Berendsen barostat [5] with a coupling time of 0.5 ps and 1 atm pressure. The production runs are performed in canonical ensemble. The electrostatics are treated using Particle Mesh Ewald [6]. The interaction cutoff is chosen as 1.4 nm.

We use PMMA chains of lengths $N_l = 30$ solvated in a simulation box consisting of 2.0×10^4 solvent molecules with varying x_c. In Supplementary Fig. we show a plot of the all-atom simulation of PMMA in aqueous methanol. We also want to point out that the case 2 in the generic model is tuned to reproduce PMMA solvation in aqueous methanol. However, the all-atom chain consists of $\sim 15\ell_p$ with ℓ_p being the presistance length of the chain, while in the generic model we have simulated a chain of $30\ell_p$ length. If we now take $R_{\rm g}$ for the maximally swollen chain $R_{\rm g}$ and normalized it by $(N_l/\ell_p)^{1/3}$ taking a collapsed chain, we find $R_{\rm g}\,(N_l/\ell_p)^{-1/3} = 0.67\sigma$ for the generic model and 0.59 nm for all-atom chain. This gives a conversion of $1\sigma \sim 0.9$nm between all-atom and generic simulation.

Supplementary Note 2: Shift in chemical potential

In order to further consolidate the relevance of the generic model for explaining polymer swelling in poor solvent mixtures, we now investigate the thermodynamic consistency between the all-atom data and the generic simulations. It is important to mention that the solvation of macromolecules is intimately linked to the energy density within the solvation volume, which is dictated by the relative interaction strengths between monomers and (co)solvent components and the relative size of (co)solvents with respect to the monomer size. In our definition thermodynamic consistency is referred to when we reproduce correct solvation energy (or chemical potential) between two models. Here, we calculate chemical potential μ_p using the Kirkwood-Buff theory of solutions [7, 8]. If p at a dilute concentration is solvated in a mixture of s and c, μ_p can be calculated using [9],

$$\left(\frac{\partial \overline{\mu}_p}{\partial \rho_c}\right)_{p,T} = \frac{\mathrm{G}_{ps} - \mathrm{G}_{pc}}{1 - \rho_c(\mathrm{G}_{cs} - \mathrm{G}_{cc})}, \tag{1}$$

where $\overline{\mu}_p = \mu_p/k_\mathrm{B}T$, and ρ_c is the cosolvent number density. G_{ij} is the Kirkwood-Buff integral that is related to the pair distribution function $\mathrm{g}_{ij}(r)$ via $\mathrm{G}_{ij} = 4\pi\int_0^\infty [\mathrm{g}_{ij}(r) - 1]\, r^2 dr$. The integration of Supplementary Eq. 1 gives a direct estimate of the shift in $\overline{\mu}_p$ with increasing x_c.

In Supplementary Fig. we show $\overline{\mu}_p$ as a function of x_c. The shift in $\overline{\mu}_p$ per monomer between solvent and cosolvent is $\sim 2k_\mathrm{B}T$, i.e. $f(N_l)\overline{\mu}_p/N_l \sim 2k_\mathrm{B}T$. Considering $f(N_l) \sim 2$, this leads to $\left[\mu_p(x_c = 0) - \mu_p(x_c = 1)\right]/N_l \sim k_\mathrm{B}T$ for polymers. It is also clear that the generic model correctly reproduces intermolecular affinities and its description within the standard Flory-Huggins theory.

$$\begin{aligned} \bar{\mu}_p\left(\phi_p \to 0\right) &= const - x_c \ln x_c - (1 - x_c)\ln(1 - x_c) \\ &+ (1 - x_c)\chi_{ps} + x_c\chi_{pc} \\ &- 2x_c\left(1 - x_c\right)\chi_{sc}. \end{aligned} \tag{2}$$

[1] K. Murzyn, M. Bratek, and M. Pasenkiewicz-Gierula J. Phys. Chem. B **117**, 16388 (2013).
[2] H. J. C. Berendsen, J. R. Grigera, and T. P. Straatsma, J. Phys. Chem. **91**, 6269 (1987).
[3] W. L. Jorgensen, D. S. Maxwell, and J. Tirado-Rives, J. Am. Chem. Soc. **118**, 11225 (1996).
[4] D. Mukherji, B. Baumeier, and K. Kremer, in preparation.
[5] H. J. C. Berendsen, J. P. M. Postma, W. F. van Gunsteren, A. DiNola, and J. R. Haak, J. Chem. Phys. **81**, 3684 (1984).
[6] U. Essmann, L. Perera, M. L. Berkowitz, T. Darden, H. Lee, L. G. A. Pedersen, J. Chem. Phys. **103** 8577 (1995).
[7] J. G. Kirkwood and F. P. Buff, J. Chem. Phys. **19**, 774 (1951).
[8] A. Ben-Naim, J. Phys. Chem. **71**, 4002 (1967).
[9] J. Rösgen, B. M. Pettitt, and D. W. Bolen, Biophys. J **89**, 2988 (2005).
